# The effect of iron on Chilean *Alexandrium catenella* growth and paralytic shellfish toxin production as related to algal blooms

**DOI:** 10.1007/s10534-021-00349-2

**Published:** 2021-10-30

**Authors:** Kyoko Yarimizu, Jorge I. Mardones, Javier Paredes-Mella, Luis Norambuena-Subiabre, Carl J. Carrano, Fumito Maruyama

**Affiliations:** 1 Microbial Genomics and Ecology, Office of Academic Research and Industry-Government Collaboration, Hiroshima University, 1-3-2 Kagamiyama, Higashi-Hiroshima City, Hiroshima 739-8511 Japan; 2 Centro de Estudios de Algas Nocivas (CREAN), Instituto de Fomento Pesquero (IFOP), Padre Harter 547, 5480000 Puerto Montt, Chile; 3 Centro FONDAP de Investigación en Dinámica de Ecosistemas Marinos de Altas Latitudes (IDEAL), Valdivia, Chile; 4 Department of Chemistry and Biochemistry, San Diego State University, 5500 Campanile Dr., San Diego, CA 92182-1030 USA

**Keywords:** Trace metal, Iron, *Alexandrium catenella*, Paralytic shellfish toxin (PST), Harmful algal bloom (HAB)

## Abstract

The dinoflagellate *Alexandrium catenella* is a well-known paralytic shellfish toxin producer that forms harmful algal blooms (HABs) worldwide. Blooms of this species have repeatedly brought severe ecological and economic impacts to Chile, especially in the southern region, where the shellfish and salmon industries are world-famous. The mechanisms of such HABs have been intensively studied but are still unclear. Nutrient overloading is one of the often-discussed drivers for HABs. The present study used the *A. catenella* strain isolated from southern Chile to investigate how iron conditions could affect their growth and toxin production as related to HAB. Our results showed that an optimum concentration of iron was pivotal for proper *A. catenella* growth. Thus, while excess iron exerted a toxic effect, low iron media led to iron insufficiency and growth inhibition. In addition, the study shows that the degree of paralytic shellfish toxin production by *A. catenella* varied depending on the iron concentration in the culture media. The *A. catenella* strain from southern Chile produced GTX1-4 exclusively in the fmol cell^−1^ scale. Based on these findings, we suggest that including iron and paralytic shellfish toxin measurements in the fields can improve the current HAB monitoring and contribute to an understanding of *A. catenella* bloom dynamics in Chile.

## Introduction

*Alexandrium catenella* is one of the most prominent toxin-producing phytoplankton species and has been increasingly detected on many coasts in the world for decades (Penna et al. 2005; Persich et al. 2006; Anderson et al. 2012). In Chile, *A. catenella* was first detected in 1972 in the Magallanes region, reached the Aysén region in 1992, and was further expanded to the southern Chiloé Island in the Los Lagos region in 2002 (Guzmán et al. 1975, 2002; Molinet et al. 2003; Varela et al. 2012). Subsequently, *A. catenella* was detected for the first time on the offshore Pacific coast of Chiloé Island in 2009 and further north along the Pacific coast of the Los Rios region in 2016 (Mardones et al. 2010; Buschmann et al. 2016; Paredes et al. 2019). Due to the paralytic shellfish toxins (PST) that *A. catenella* produces, blooms of this species in Chile have repeatedly brought severe ecological and economic impacts, especially in the southern region where the shellfish and salmon industries are world-famous (Molinet et al. 2003; Mardones et al. 2010; Varela et al. 2012; Díaz et al. 2019). For instance, the *A. catenella* bloom that occurred in the Aysén region in 2002 caused over 50 human intoxications, three fatalities, and the loss of 1800 metric tons of farmed salmon (Fuentes-Grünewald et al. 2008). The Los Lagos region experienced *A. catenella* blooms in 2006 and 2009, with damages on the shellfish industry and fish kills equivalent to US$ 9.2 and 10 million, respectively (Fuentes-Grünewald et al. 2008; Mardones et al. 2010, 2015). The *A. catenella* bloom in 2016 was particularly severe, affecting 600 km of benthic artisanal fisheries and over 200 shellfish farms in the Los Lagos region, resulting in economic damage to the coastal communities and igniting social protests that lasted three weeks (Trainer et al. 2020). In 2018, another intense *A. catenella* bloom event reached a world record of 143,130 µg saxitoxin (STX) in 100 g of shellfish flesh from the Aysén Region, while the regulatory limit is 80 μg STX in 100 g of flesh (Álvarez et al. 2019). It is clear that impacts on the Chilean coastal waters and communities by the blooms of *A. catenella* have been increasing over the past decade (Paredes et al. 2019, 2020).

Elucidating the mechanisms leading to Harmful Algal Blooms (HABs) has been a challenging task for a long time. Nutrient overload is a frequently discussed topic as a cause of HAB, and those studies revolve around the potential role of macronutrients; nitrogen, phosphorus, and silicate (Hallegraeff 1993; Paredes et al. 2020). In fact, many current algal monitoring programs include nutrient analysis to investigate the effect of these nutrients on bloom dynamics (Hallegraeff and Gollasch 2006). However, iron can also be a growth controlling factor for phytoplankton. Even though iron is one of the most abundant elements on the Earth, it is poorly bioavailable due to its extreme insolubility but remains a crucial nutrient for all living organisms, including phytoplankton (Martin and Fitzwater 1988; Wu and Luther 1994). Under the aerobic and mildly alkaline oceanic conditions, the predominant iron state is largely insoluble Fe(III), with the little soluble Fe(III) mostly complexed with natural organic ligands, which leave negligible bioavailable iron to the surroundings (Rue and Bruland 1995; Martin and Fitzwater 1988; Bruland et al. 1991; Wu and Luther 1994; Sunda 2006). The profound effects of low iron concentrations on phytoplankton growth can be seen in the oceans’ so-called High Nutrient Low Chlorophyll (HNLC) regions. The HNLC regions are defined by poor phytoplankton growth despite highly available “major nutrients” but near negligible iron concentrations (Martin and Fitzwater 1988; Martin et al. 1994; Coale et al. 1996; Boyd et al. 2000; Maldonado et al. 2005). Brzezinski et al. (2015) also measured oceanic iron together with other nutrient in California Current, reporting that phytoplankton growth in low-Si: N, high-N: Fe waters responded strongly to added iron, confirming growth limitation by iron. Nevertheless, the relationship between HAB species and iron has been far less studied than that between HAB species and the “major nutrients” such as nitrogen, phosphorus, and silicate. The HAB monitoring that includes iron measurements, as exemplified in Brzezinski et al. (2015), has not been documented in Chile to date. One of the primary reasons for it is that the electrochemical measurement of iron in seawater is a complex and challenging task because of its low concentrations, its existence in two redox states, Fe(II) and Fe(III), as well as the potential contamination by ferrous containing apparatus (Croot and Heller 2012).

So far, little information is available on iron concentration in Chilean waters. A study measured iron in transect between the Marquesas Islands to the Chilean coast and reported that total dissolved iron in the Chilean upwelling ranged between 1.2 and 3.9 nM (Blain et al. 2008). This group also reported that onshore water in a southern Chile station (− 34.5471, − 72.4047) contained 1.2 nM total dissolved iron at 30-m depth. However, iron concentration in other Chilean water regions is unknown, and how iron influences *A. catenella* blooms in Chile is difficult to predict. In general terms, iron concentrations in the oceans are at nanomolar levels (Bruland et al. 1991). Iron typically increases with water depth in vertical columns, as iron in the photic zone is used for primary production (Vraspir and Butler 2009). For example, the offshore Peruvian waters between 9 to 16 ºS were reported to contain total dissolved iron of ~ 2 nM at 1000-m depth and < 1 nM at the surface (Rapp et al. 2019). The open ocean water at 1,000-m depth in the south of Australia contained 0.25 – 0.37 nM total dissolved iron, while that in the upper mixed layer varied between 0.2 and 0.4 nM (Ibisanmi et al. 2011). Regarding the horizontal comparison across the surface ocean water, total dissolved iron depends on location. For example, a group that measured iron at a transect covering the entire western Atlantic Ocean showed that the total dissolved iron in surface water was < 4 nM depending on location (Rijkenberg et al. 2014). From the general information on oceanic iron and phytoplankton stated above, we assumed that iron could influence Chilean *A. catenella* blooms.

To understand the relationship between iron and Chilean *A. catenella*, the present study investigated the effects of iron on the growth of the *A. catenella* isolated from southern Chile and its PST production. The iron experiment was designed to observe culture *A. catenella* growth in different iron concentration media. The study also monitored cultured *A. catenella* growth at varying iron concentration changes. Further, this study was designed to quantitate saxitoxins (STX, dc-STX, neo-STX, NEO), gonyautoxins (GTX1-5, dc GTX2-3), and N-sulfocarbamoyl toxins (C1-2) by *A. catenella* grown in culture containing different iron concentrations. Based on these results, we discussed the potential roles of iron on *A. catenella* bloom dynamics.

## Materials and methods

### Growth media

All plastic and glass containers were soaked in 3 N hydrochloric acid for two weeks, rinsed with Milli-Q water, and dried in a laminar-flow air bench before use to eliminate any iron contamination (Bruland et al. 1979). Seawater (SW) from Metri (− 41.597, − 72.7056, Los Lagos, Chile) was filtered through a 0.22-μm pore-sized membrane (MilliporeSigma, WHA7402004), mixed with 0.005% hydrochloric acid (Trace metal-free HCl, Fisher Scientific, A466-250), and autoclaved at 121 ºC for 30 min. The autoclaved SW was maintained at a salinity of 30 and pH range between 8.0 and 8.2 at ambient temperature. Sterile L1 nutrient and trace metal mix without FeCl_3_/Na_2_EDTA were prepared according to Guillard and Hargraves (1993). L1 and L1 without Fe were added to the sterile SW to make a growth media with targeted total iron concentrations from 0 to 10,000 nM. It should be noted that the media included all iron phases, and total “dissolved iron” concentrations in the media are much lower than the total iron concentrations ([Fe]_T_). Measuring total dissolved iron concentrations in the media is not possible because equilibrium between the various iron species at pH 8.2 is reached extremely slowly, resulting in unstable total dissolved iron measurements.

### Algal isolation and maintenance

*Alexandrium catenella* strain *CREAN AC11* was isolated from a cyst collected from Quellón (Los Lagos, Chile) and used for all experiments herein. Quellón is one of the most affected areas by *A. catenella*. The process of *A. catenella* cell isolation was as follow: Sediment samples were collected by scuba divers from Quellón in 2014. The sediment samples were transported to the laboratory, where the cysts of *A. catenella* cysts were isolated using the methodology described by Varela et al. (2012). The cysts were then placed in multi-welled cultivation plates with SW + L1 medium and exposed to conditions to facilitate germination (i.e., 12 °C, 30 psu, 35 mmol photons m^−2^ s^−1^, and a photoperiod of 16:8 h light/dark). From each well where cysts germinated, a single cell was transferred to a new well (48-well plate, area of 0.64 cm^2^ well^−1^) using an inverted microscope (Olympus CKX 42) and extended Pasteur pipettes (Andersen and Kawachi 2004). Each well was filled with 500 ml of SW + L1 growth media and verified the successful transfer of single cells using an inverted microscope. When the single cell began its division and cell count was increased, cells from each well were transferred into SW + L1 in sterile containers (Nunc cell culture treated flasks with filter caps, Fisher Scientific, 12-565-57). Cultures were maintained under 50 μmol photons m^−2^ s^−1^ on 16:8 h light: dark cycle at 15 ± 2 °C (standard growth condition). The upper portion of a culture containing healthy cells was diluted to < 500 cells ml^−1^ with new SW + L1 media every three weeks. Different strain names were given to different cysts. After screening several strains, the strain *CREAN AC11* was used in this study as it was one of the most viable strains.

### Growth of A. catenella in controlled iron media

Culture was pretreated with 0.1% (v/v) antibiotics (penicillin 5units ml^−1^, streptomycin 5 μg ml^−1^, and neomycin 10 μg ml^−1^) (Sigma Aldrich, P4083-100 ml) for 24 h under the standard growth condition. This was transferred into 15-ml sterile tubes and centrifuged at 12,750 m s^−2^ for five seconds to collect cell pellets. The supernatant was removed by inversion, and cell pellets were washed three times with SW. Then, cells were re-suspended with SW + L1 containing the target total iron concentrations, [Fe]_T_ = 0, 10, 100, 1000, and 10,000 nM. Cultures were maintained under the standard growth condition and monitored for the cell count using microscopy every 4–6 days for two consecutive subcultures. All data were collected triplicate.

### Rescuing iron deficient A. catenella

*Alexandrium catenella* culture in SW + L1 media was aliquoted in two separate containers. One was maintained in SW + L1 as a control (containing [Fe]_T_ = 10,000 nM) and subsequently cultured with SW + L1 every three weeks, while the other was subsequently diluted with SW + L1 without Fe media every three weeks. Therefore, the first, second, and third subcultures were cell growth comparisons between the control and cultures in SW + L1 with [Fe]_T_ = 1300 nM, 468 nM, and 100 nM, respectively. [Fe]_T_ = 5000 nM was added to the third subculture containing [Fe]_T_ = 100 nM on day-31, and the growth was monitored for additional 22 days. All data were collected triplicate.

### Growth responses

Growth rates (GR) were determined through a linear regression model γ i = α + βχi (Guillard and Hargraves 1993), where γ i = ln-transformed cell density (cells ml^−1^), χi = time (days), α = intercept, and β = growth rate (cell division day^−1^). Cell density at the end of exponential phase was used as the maximum cell density response (MCD) in cells ml^−1^.

### Statistical analyses

Analyses of variance (ANOVA) were performed in the linear model framework to evaluate the effect of iron concentration variation (first and second subculture experiments), the effect of iron reduction, or iron addition (rescue experiment) on the GR and MCD responses. A post hoc Tukey HSD analysis was performed to evaluate pairwise growth differences among treatments (levels) of the main effects. In each case, the null statistical hypotheses were rejected at a significance level (α) of 0.01. Analyses were performed with R software (R Core Team 2020).

### Paralytic shellfish toxin assay

The method from Ravn et al. (1995) was optimized. *Alexandrium catenella* was grown in SW + L1 containing [Fe]_T_ = 100, 1000, and 10,000 nM. The condition of [Fe]_T_ = 0 and 10 were not used for toxin assay because *A. catenella* did not grow under these conditions. On day-7 and day-14, cell counts were recorded, and 50 ml of each culture was filtered through GF/F membranes (MilliporeSigma, WHA1825047), which were cut into pieces and soaked in 1.2 ml of diluent (acetic acid 0.05 M). The samples were vortexed and sonicated for 10 min followed by filtration through a 0.2 μm pore-sized membrane (MilliporeSigma, WHA67771302) to collect filtrate containing toxins. The filtrate was diluted to 1/4 with acetonitrile and analyzed by Hilic-UHPLC-MS/MS per method by Turner et al. (2019): QQQ Mass Spectrometer (Agilent, G6240A), UHPLC (Agilent, 1290 Infinity) consisting of a quaternary pump G7104A, a high-performance autosampler G7129B, and a column oven G7130A, column (Acquity UHPLC BEH amide column, 100 × 2.1 mm, 1.7 µm particle size), injection volume of 2 µL, capillary voltage of 3 kV in the negative mode and 3.5 kV in the positive mode, gas flow of 12 l min^−1^ at 350 °C, and nebulizer pressure of 35 psi. The chromatographic conditions and mrm transitions for the different PST analogs were as described in Turner et al. (2019). Certified reference toxins for PST were obtained from the National Research Council of Canada (NRCC, Halifax, Canada). Data processing was done with MassHunter software to provide saxitoxins (STX, dc-STX, neo-STX, NEO), gonyautoxins (GTX1-5, dc GTX2-3), and N-sulfocarbamoyl toxins (C1-2).

## Results

### Effect of iron on A. catenella growth

**Fig. 1:**
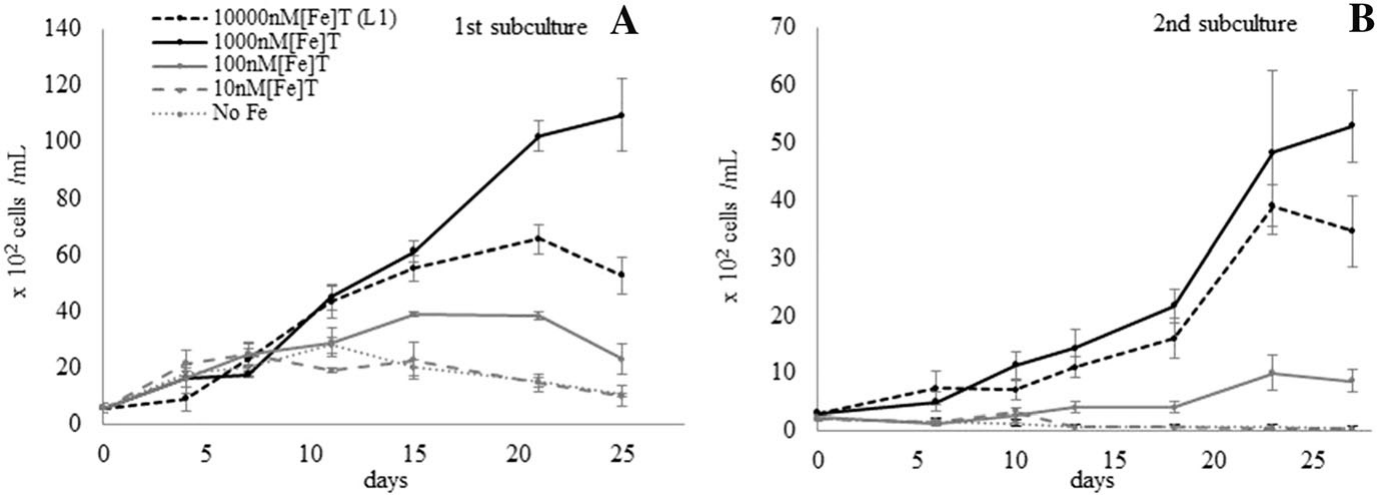
*Alexandrium catenell*a strain *CREAN AC11* in culture media with different total iron concentrations [Fe]_T_: The growth media was SW + L1 with [Fe]_T_ of 10,000, 1000, 1000, 10, or 0 nM. The *A. catenella* in **A** the first subculture and **B** second subculture were grown under the standard growth condition

The growth of *A. catenella* was compared in the SW + L1 containing five different [Fe]_T_ (10,000, 1000, 100, 10, and 0 nM) for the first and second subcultures (Fig. 1). The cells in the media containing [Fe]_T_ = 1000 nM grew the greatest in both subcultures, in which the GR for the first and second subculture was 0.137 and 0.120 cell division day^−1^, and the MCD was 10,933 and 4833 cells ml^−1^, respectively (Table 1). Those in the media containing [Fe]_T_ = 10,000 nM showed the second most significant response, in which the GR for the first and second subculture was 0.127 and 0.103 cell division day^−1^, and the MCD was 6567 and 3900 cells ml^−1^, respectively (ANOVA p < 0.01, Table 1). On the contrary, the cells in the media containing [Fe]_T_ = 100 nM showed a very slow response in both first and second cultures, in which the GR was 0.084 and 0.069 cell division day^−1^, and the MCD was 3900 and 1000 cells ml^−1^, respectively. Those in the media containing [Fe]_T_ ≤ 10 nM showed the worst growth in both subcultures (ANOVA p < 0.01, Table 1).

**Table 1:** Statistical analysis of *A. catenella* strain *CREAN AC11* growth rate in various iron conditions

[Fe]_T_	GR	SD	Tuk.	MCD	SD	Tuk.	Attribute
10,000	0.127	0.007	c	6567	513	b	Figure 1A
1000	0.137	0.019	c	10,933	1286	c	
100	0.084	0.012	b	3900	100	a	
10	0.029	0.019	a	2467	416	a	
0	0.035	0.008	a	2800	265	a	
[Fe]_T_	GR	SD	Tuk.	MCD	SD	Tuk.	Figure 1B
10,000	0.103	0.014	b	3900	361	b	
1000	0.120	0.014	b	4833	1422	b	
100	0.069	0.002	b	1000	300	a	
10	− 0.170	0.011	a	333	153	a	
0	− 0.118	0.085	a	133	58	a	
[Fe]_T_	GR	SD	Tuk.	MCD	SD	Tuk.	Figure 2A
10,000 (control)	0.110	0.022	b	2900	2900	a	
1300	0.088	0.013	b	1900	1900	a	
10,000 (control)	0.089	0.007	b	6533	6533	b	Figure 2B
468	0.045	0.013	a	2533	2533	a	
[Fe]_T_	GR	SD	Tuk.	MCD	SD	Tuk.	Figure 2C
10,000 (control)	0.103	0.014	a	3900	3900	b	
100	0.069	0.002	a	1000	1000	a	
Rescue	0.222	0.041	b	4733	4733	b	

The list shows Growth rate (GR) in cell division day^−1^, maximum cell density (MCD) in cells ml^−1^, standard deviation (SD), and results of post hoc Tukey HSD (Tuk) after ANOVA per experiments. [Fe]_T_ denotes the total iron concentration in nM corresponding to the attributes. The GR and MCD were evaluated independently, and each group (divided by the border lines in Table) was evaluated separately. The control was SW + L1 which contained [Fe]_T_ = 10,000 nM

### Rescuing iron-starved A. catenella

**Fig. 2:**
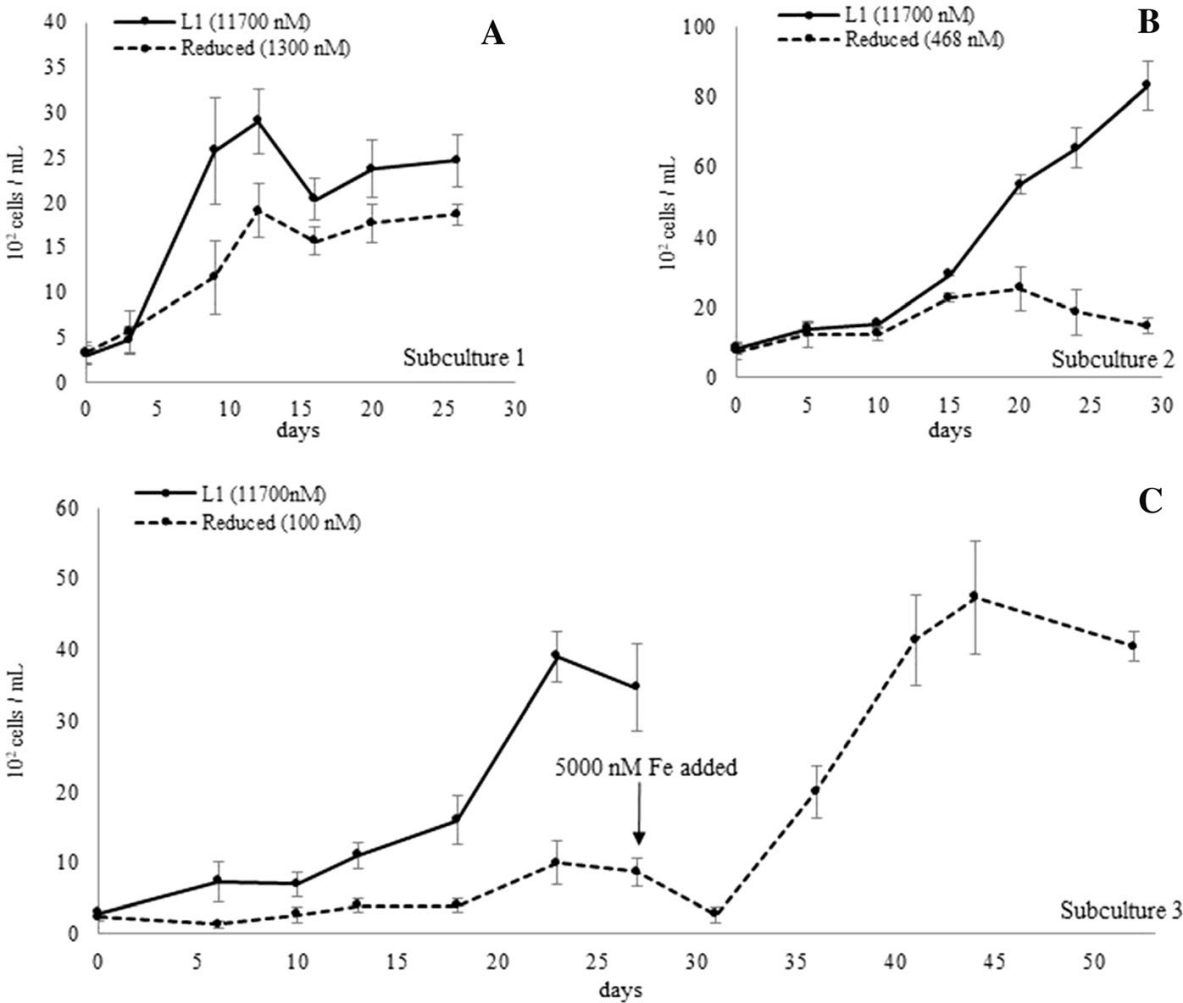
Growth of *A. catenella* strain *CREAN AC11* in progressive diminution of total iron concentration and iron supplement effect on iron-deficient *A. catenella*: The growth of *A. catenella* maintained in **A** the first subculture in SW + L1 with [Fe]_T_ = 1300 nM, **B** second subculture in SW + L1 with [Fe]_T_ = 468 nM, **C** third subculture in SW + L1 with [Fe]_T_ = 100 nM. The control was *A. catenella* in SW + L1 which contained [Fe]_T_ = 10,000 nM. The *A. catenella* growth after iron supplement of [Fe]_T_ = 5000 nM was given as shown in (**C**)

In the progressive diminution of total iron concentration experiment, the first, second, and third subculture contained SW + L1 with [Fe]_T_ = 1300 nM, 468 nM, and 100 nM, respectively. The growth of *A. catenella* in the media with [Fe]_T_ = 1300 nM was comparable to that of control containing L1 level iron concentration (≒10,000 nM) (Fig. 2A), in which the GR of [Fe]_T_ = 1300 nM culture and that of control in the first, second, and third subculture was 0.088, 0.110, 0.089, and 0.103 cell division day^−1^, respectively (ANOVA p < 0.01, Table 1). The cell growth in the second subculture with [Fe]_T_ = 468 nM notably slowed down (Fig. 2B), as the GR was 0.045 cell division day^−1^ (ANOVA p < 0.01, Table 1). By the third subculture where [Fe]_T_ = 100 nM, *A. catenella* cells were exposed to the iron-limited condition for three consecutive subcultures (appx. 3 months), the cells struggled to grow, as the GR was 0.069 cell division day^−1^ and MCD was 1000 cells ml^−1^. Given [Fe]_T_ = 5000 nM to this iron-limited culture resulted in the rapid recovery of GR to 0.222 cell division day^−1^ with MCD of 4,733 cells ml^−1^ (Fig. 2C). There was no statistical difference between the control and [Fe]_T_ = 5000 nM cultures for MCD (ANOVA p > 0.01, Table 1), indicating that the cells had ceased growing under iron deficiency but were rescued by the iron addition.

### Iron effect on A. catenella PST production

The *A. catenella* in SW + L1 with [Fe]_T_ = 100, 1000, and 10,000 nM were analyzed on day-7 and day-14 for production of PST, specifically saxitoxins (STX, dc-STX, neo-STX, NEO), gonyautoxins (GTX1-5, dc GTX2-3), and N-sulfocarbamoyl toxins (C1-2). All samples showed the presence of GTX1-4 and the absence of other tested toxins (Table 2). Among gonyautoxins, GTX-3 was the most concentrated toxin in all samples. For example, a cell in the media containing [Fe]_T_ = 1000 nM on day-7 contained 9.3, 13.9, 50.7, and 15.9 fmol cell^−1^ of GTX-1, GTX-2, GTX-3, and GTX-4, respectively. The total PST production by an *A. catenella* cell was pronounced the most at [Fe]_T_ = 1000 nM at day-7: On day-7, the total PST was 49.8, 89.8, and 71.8 fmol cell^−1^ for the condition containing [Fe]_T_ = 100, 1000, and 10,000 nM, respectively. On day-14, the total PST was 12.9, 33.6, and 10.4 fmol cell^−1^ for the condition containing [Fe]_T_ = 100, 1000, and 10,000 nM, respectively.

**Table 2:** PST concentration per *A. catenella* cell

Day	[Fe]_T_	GTX 1	GTX 2	GTX 3	GTX 4	Total toxin
7	100 nM	4.1 (1.6)	9.3 (0.4)	27.8 (9.5)	8.6 (1.8)	49.8
	1000 nM	9.3 (1.5)	13.9 (0.4)	50.7 (11.6)	15.9 (0.1)	89.8
	10,000 nM	7.7 (3.5)	10.4 (0.1)	41.3 (6.7)	12.4 (0.3)	71.8
14	100 nM	1.2 (0.1)	1.9 (0.5)	8.4 (4.7)	1.4 (0.2)	12.9
	1000 nM	2.3 (1.1)	2.3 (0.1)	20.1 (2.0)	8.9 (2.8)	33.6
	10,000 nM	1.3 ± 0.2	2.1 ± 0.4	5.5 ± 1.4	1.6 ± 0.1	10.4

The *A. catenella* was grown in the SW + L1 containing [Fe]_T_ = 100, 1000, and 10,000 nM. At day 7 and 14, 50-ml of each culture was filtered and analyzed for PST. Saxitoxins (STX, dc-STX, neo-STX, NEO), gonyautoxins (GTX5, dc GTX2-3), and N-sulfocarbamoyl toxins (C1-2) were tested but not detected. No peak was detected from diluent. The unit in the table is fmol cell^−1^. Numbers in parentheses are standard deviation in plus/minus (n = 3). Total toxin is summation of mean values.

## Discussion

This study presented evidence that iron was an essential element and growth control nutrient for the Chilean *A. catenella* strain *CREAN AC11*. In fact, iron addition rescued poorly growing *A. catenella* cells suffering from iron deficiency. There was, however, an optimum iron condition for *A. catenella*, which appeared to be [Fe]_T_ = 1000 nM. Excess iron exerted a toxic effect, and insufficient iron completely inhibited cell growth for this species. This is consistent with the behavior of most trace elements on biological systems, where nutrient metals have optimal concentrations for a target species, above which growth is limited by intoxication, and below which growth is inhibited by deficiency (Sunda 2006). Therefore, including field iron concentration measurement, likely by anodic stripping voltammetry analysis, to current HAB monitoring programs can be beneficial (Gledhill and van den Berg 1994; Rue and Bruland 1995; van den Berg 1995; Yarimizu et al. 2019). Detection of iron concentration changes in the local marine environment may contribute to early warning of *A. catenella* blooms.

This study demonstrated that the *A. catenella* cellular toxin production was a fmol cell^−1^ scale and was most pronounced in the media containing [Fe]_T_ = 1000 nM, the optimum condition for their growth, at the beginning of the exponential growth phase. This toxin scale is consistent with prior studies, reporting a wide range of toxin content (0.1 to 450 fmol cell^−1^) for *A. catenella* species but all in the fmol scale per cell (Krock et al. 2007; Laabir et al. 2013). Corresponding to our observation, He et al. (2010) performed a similar experiment with *Alexandrium tamarense* isolated from the sediment in Hong Kong coastal area, reporting that iron availability controlled the growth and toxin production of this species. However, their result was different in which the toxin production by *A. tamarense* was most enhanced under iron concentration of 10,000 nM (He et al. 2010).

The present study further showed that *A. catenella CREAN AC11* produced GTX 1–4 exclusively. This is consistent with the report by Carbonell et al. (2016) who tested *A. catenella* obtained from the same region, resulting in exclusive GTX 1–4 production. The toxin production seems to be strain-specific, as many studies reported that *Alexandrium* species produced different types of PST depending on geographic origin and environmental factors such as salinity (Hamasaki et al. 2001; Grzebyk et al. 2003; Lim and Ogata 2005; Etheridge et al. 2005), temperature (Anderson et al. 1990; Etheridge et al. 2005), light intensity (Hwang and Lu 2000; Etheridge et al. 2005), and nutrients (Poulton et al. 2005; Xin et al. 2010). The documented PST in southern Chilean *A. catenella* strains were C1, C2, B1, GTX1, and GTX4 (Krock et al. 2007), C2, GTX3, GTX4, and neoSTX (Varela et al. 2012), and neoSTX, GTX1-5 (Aguilera-Belmonte et al. 2013). Those in the East China Sea, Hong Kong, and France were C1-2 (Li et al. 2011), GTX-1, GTX-3, and GTX-4 (Siu et al. 1997; Xu et al. 2012), and C1-C4 plus GTX3-5 (Laabir et al. 2013), respectively. Gaining knowledge of the cellular PST contents and profiles of *A. catenella* is vital because STX is one of the most potent natural neurotoxins, and a dose of 1 mg of STX is fatal to humans (Wiese et al. 2010). In Chile, STX and its derivatives from *A. catenella* have caused hundreds of people to suffer from paralytic shellfish poisoning syndromes upon consuming contaminated shellfish (García et al. 2004; Krock et al. 2007). Routine monitoring of STX in coastal waters can provide important implications for strategizing HAB detection, toxicity warning, and PST removal from a contaminated source.

While this laboratory study supported the hypothesis that iron controls Chilean *A. catenella* growth and toxin production, it is unclear how it operates in the field. It is one of our next assignments to collect iron data from Chilean waters involving both HAB and non-HAB bloom events.

The water in southern Chile has a strong influence from freshwater, glaciers, and coastal runoff in different proportions according to the proximity of the freshwater sources (Sievers and Silva 2008). Freshwater generally contains high iron, and how much river water flows into the sea varies depending on seasons and locations. To best of our knowledge, there is no documented data on iron concentration in either Chilean seawater or freshwater to date. However, other areas typically have ng l^−1^ for seawater iron and mg l^−1^ for freshwater. For example, the average total iron in coastal seawater of Southern California and Baja California was around 5 ng l^−1^ (Yarimizu et al. 2019). The average total iron in freshwater at Perhonjoki River, Finland, was reported 1.5–3 mg l^−1^, which was increased to 4–6 mg l^−1^ after heavy rains and during snowmelt period (Myllynen et al. 1997). Therefore, southern Chilean coast consisted of fjords can be influenced by iron concentration fluctuation depending on nearby freshwater’s input. In addition, the open ocean water in the South America is a patch of shallow waters, which can be another factor for the upwelling regime in the region (Hutchins et al. 2002). Those factors could easily alter the local seawater’s properties, leading it to interchange the iron redox stages and ratios and creating the regional Fe-limited and Fe-replete areas that potentially influence the *A. catenella* blooms. For the above stated reasons, investigating a correlation between rainfall, seasons, and iron concentration may provide information to characterize the local HABs.

In the meantime, the possibility of anthropogenic factors as drivers of *A. catenella* blooms in southern Chile cannot be eliminated. The salmon industry in southern Chile has rapidly expanded the scale of businesses since the 1980s to its current production of nearly 800,000 tons per year (Mascareño et al. 2018; Armijo et al. 2020). It is a constant concern for the locals that the nutrient overloading to the coastal waters to feed salmon could change the ecosystem. For the HAB events in 2016 in Chile, a precursor bloom of *Pseudochattonella verruculosa* killed tons of salmon in northern Chiloé Island first, and the dead salmon were dumped off the Islands’ coast with the government’s permission. The notorious *A. catenella* bloom occurred in the region soon after (Mascareño et al. 2018; Armijo et al. 2020). The Chilean government claimed that El Nino was the cause of this *A. catenella* bloom (Buschmann et al. 2016; Armijo et al. 2020; Trainer et al. 2020). However, a debate between the government and locals is ongoing whether the cause was anthropogenic eutrophication. Salmon is an iron-rich food source (0.8 mg iron per 100 g flesh of wild Atlantic raw salmon, USDA FDC ID 173086, NDB 15076). Theoretically, throwing 4700 tons of dead salmon could inject massive iron into the local ocean, which could leave speculation that this action may have influenced phytoplankton biomass increase, as exemplified in the open-ocean iron enrichment experiment (IronEX I) by Martin et al. (1994).

Lastly, it should be outlined that this study focused on a southern Chilean *A. catenella* strain. The obtained toxin profile and response to iron concentration can be different for the strains from other regions. For instance, Paredes et al. (2020) evidenced that the growth responses of *A. catenella* clones isolated along Chilean fjords have different reaction norms under different drivers. It is one of the reasons that each HAB is unique and unpredictable.

## Conclusion

This study demonstrated that iron is a growth-limiting and -stimulating factor for the Chilean *A. catenella* strain *CREAN AC11.* The study also revealed that the toxins detected from this strain were GTX1-4 in fmol scale per cell, and its production was increased with the optimum iron concentration in media. Accounting for these findings, we suggest that the Chilean HAB monitoring may improve if the measurement of iron concentration and saxitoxin in water samples are included. The information can help understand the environmental drivers for the *A. catenella* bloom dynamics, with the concomitant mitigation on socioecological and economic issues related to the HABs.

## Appendix

See Table 3.

**Table 3:** List of materials and reagents used to prepare nutrient for media preparation

Manufacture	Item	Part number
Sigma–Aldrich	Iron(III) chloride hexahydrate	10025-77-1
	Manganese(II) chloride tetrahydrate	13446-34-9
	Hexadecyltrimethylammonium bromide	H6269-100G
	Zinc sulfate heptahydrate	1088830500
	Cobalt(II) chloride hexahydrate	8025400010
	Copper(II) sulfate pentahydrate	209198-5G
	Sodium molybdate dehydrate	331058-5G
	Selenous acid	211176-10G
	Nickel(II) sulfate hexahydrate	227676-100G
	Sodium orthovanadate	450243-10G
	Potassium chromate	216615-100G
	Ethylenediaminetetraaceticacid, tetrasodium salt dihydrate	BP121-500
